# Commentary: Attention to Eyes Is Present but in Decline in 2–6-Month-Old Infants Later Diagnosed with Autism

**DOI:** 10.3389/fpubh.2015.00272

**Published:** 2015-12-08

**Authors:** Jennifer C. Sarrett, Karen S. Rommelfanger

**Affiliations:** ^1^Center for the Study of Human Health, Emory University Atlanta, Atlanta, GA, USA; ^2^Center for Ethics Neuroethics Program, Emory University Atlanta, Atlanta, GA, USA; ^3^Department of Neurology, Emory University Atlanta, Atlanta, GA, USA; ^4^Department of Psychiatry and Behavioral Sciences, Emory University Atlanta, Atlanta, GA, USA

**Keywords:** pediatric ethics, neuroethics, autism spectrum disorder, eye-tracking, preclinical detection, early detection technology

A recent *Nature* article provided preliminary evidence that infants age 2–6 months old, who were later diagnosed with Autism Spectrum Disorder (ASD), fixated more on the mouth than eyes and more at objects than people when viewing videos of typical childhood social scenes (1). While the sample was small, a reliable pattern of decline in eye fixation accurately predicted their level and classification of symptoms at age three suggesting that – for the first time – an infant could be assessed within the first 6 months of life for their potential of developing ASD (see Table 1 for studies that used eye-tracking with infants 12 months and younger). These eye-tracking devices, which are currently in clinical trials, could provide access to an affordable and objective tool with the potential for extremely early intervention. Detecting ASD risk during the first 6 months of life presents unprecedented opportunities to intervene, providing children opportunities to build critical skills before autistic characteristics fully emerge. Because the eye-tracking device allows for a non-invasive, portable assessment, the device could also enable pediatricians to provide comparable screening services globally. With such promise, a near future where infants are placed into an eye-tracking device at routine pediatric visits is compelling, if not guaranteed.

**Table 1:** **Eye-tracking studies in high-risk and ASD individuals up to 1 year of age**.

Study	*N*	Sex (F/M)	Age (years)	Earliest age finding (years)	Result	Location of recruitment
Elsabbagh et al. (2)	54	33/21	0.5–0.8	0.7	Infants who attended more to mouths in complex scenes, regardless of risk for ASD, developed better expressive language skills	London, UK
Shic et al. (3)	57	17/40	0.5	0.5	Infants later diagnosed with ASD have difficulty attending less to faces and less to areas of speaker’s faces that would provide verbal cues	New Haven, CT, USA
Jones and Klin (1)	11	0/11	0.2–2	0.2	Infants later diagnosed with ASD attend more to mouths.	Atlanta, GA, USA
Chawarska et al. (4)	49	15/34	0.5	0.5	Infants later diagnosed with ASD attend less attention to people and faces	New Haven, CT, USA
Elsabbagh et al. (5)	54	3/21	0.5–0.8	n/a	Infants, regardless of outcome, attend similarly to faces	London, UK
Young et al. (6)	58	26/32	0.5	n/a	Infants, regardless of outcome, do not differ in scanning of complex or simple scenes of eyes, mouths, and hands	Davis, CA, USA

*Studies with infants up to 1 year of age are featured. *N* represents children who are high risk for ASD or ASD who were confirmed prior to or after the testing that were included in the analysis. Only studies where eye-tracking data could be correlated with a diagnosis of ASD were included*.

Autism Spectrum Disorder is characterized by developmental differences and difficulties in social communication and interaction coupled with repetitive behaviors (7) with an estimated prevalence of 1 in 68 children in American populations (Developmental Disabilities Monitoring Network Surveillance Year 2010 Principal Investigators and Centers for Disease Control and Prevention (CDC) 2014) and a range of 1 in 333 to 1 in 86 among European nations. A global mean of 1 in 160 children (8) makes ASD a public health concern more prevalent than juvenile cancers (9) or diabetes (10). Diagnoses are generally made subjectively by assessing behavioral symptoms through parental interview and behavioral observations of the child (8, 9). Presently, clinicians are unable to reliably diagnose ASD until 12 months using the Autism Diagnostic Observation Schedule (ADOS) Toddler module (11); however, diagnosis usually happens much later. A 2014 CDC study estimated age at diagnosis using DSM IV TR classifications, which included several disorders under the category of ASDs rather than the DSM 5 model of ASD as a single diagnosis. It reported 48 months as the mean age of diagnosis for Autistic Disorder, 53 months for ASD or Pervasive Developmental Disorder-Not Otherwise Specified, and 75 months for Asperger’s syndrome (12). Widespread recommendations by professionals for early identification and intervention (8, 13, 14), along with a lack of viable community systems of long-term care (15), have driven priorities for autism research and funding toward reducing the age of diagnosis and intervention (16).

Currently, the American Academy of Pediatrics (AAP) (17) and the CDC (18) recommend community-wide, routine 18- and 24-month-old screenings for ASD. Early identification can provide the opportunity to facilitate early skill development and reduce the population of children and adults who are reliant on inadequate community resources for long-term care and opportunities for community engagement (15, 19). While this tool is in early phases, it has rapidly moved to clinical trials; ethical concerns must be considered alongside and as an integral part of the research, not only for future patients, but also for current study participants. Importantly, preclinical assessment technologies run the risk of bearing false negatives, i.e., children not identified as autistic but who receive an ASD diagnosis at a later age, and false positives, i.e., children identified as likely to be autistic who are not later diagnosed with ASD. Parents will undoubtedly want to do *something* with a positive screen. Studies have shown that parent’s uncertainty about their child’s medical diagnosis can lead to poor coping or adaptation to their child’s condition (20). Conversely, parents may be overly reassured by the preclinical assessment. For example, parents whose children receive a negative screen may assume their child will not develop ASD and miss opportunities to intervene.

In the event of a false positive, parents might invest in unnecessary expensive interventions, surveillance, and treatments as well as lead to changes in the life trajectories of the child, caregivers, and entire family. Caregivers may decide to change their financial plans and reallocate time and material resources to a child’s early intervention or care. Even after a false positive is identified, caregivers may be unable to cease looking for signs of ASD as a child ages or perhaps mistake a positive assessment with a diagnosis and although there is public recognition of the importance of ASD identification throughout the world (21), ASD is often considered an unwanted, even alarming, stigmatized condition (22, 23). Healthcare providers will need to be able to advise parents on what parents can do while delivering an appropriately cautious interpretation of such preclinical testing results.

We must also consider how clinicians should respond when parents whose children receive a positive screen inquire about interventions for their infant given the lack of evidenced based interventions for infants. Preclinical screens, that assess a phenotype that might predict ASD, but is not a key trait of the diagnosis, such eye-tracking technologies assess *risk* and are not diagnostic for ASD. This is important to emphasize to everyone from parents, study participants, and patient schedulers to insurance companies. Risk can be characterized in terms of both severity and susceptibility (24). ASD represents a diverse set of symptoms, abilities, and impairments and a variable timeline of development. It is unclear how much consistent predictive power preclinical testing will have for describing specific risks for severity, behavioral profiles, and age of symptom onset. With this ambiguity, there is significant potential for misunderstanding, resistance to a preclinical assessment, and damage to the therapeutic alliance of the families and clinicians.

Further, using a word like “risk” – understood differently among clinicians and the general public and across cultures – may not be wholly appropriate (25). The word “risk” may fail to communicate the vast range of possible phenotypic outcomes and instead place too much focus on negative outcomes. Adherents of a growing neurodiversity movement – an advocacy position that rejects the ideas that autism is unwanted and should be “cured” and, instead, acknowledges autism as a natural variant of human neurological development (26; See Box 1 for more on neurodiversity) – would resist the use of “risk” in relation to ASD (28). Healthcare providers and parents will need to understand the meaning of “risk” associated with a positive preclinical assessment and be able to weigh the potential benefits of treatment against the consequences of not seeking or participating in recommended interventions. Detailed communication guidelines need to be developed and disseminated with this tool alongside a public health campaign.

Box 1 **Neurodiversity and this article’s language.**

Neurodiversity is a growing advocacy movement that aims to decrease autism-related stigma by encouraging tolerance of neurological difference (27, 28). Various strategies are encouraged towards this aim, including increasing the visibility of autistic individuals throughout communities, more funding allocation for research focussed on improving the quality of life of autistic individuals (rather than “curing autism”), and inclusion of autistic voices in research, policy, and advocacy. Language is an important tool for neurodiversity. This includes using the phrase “autistic person” rather than “person with autism” to demonstrate that autism is important to identity (27). Linguistic models of neurodiveristy also includes a shift from a “language of deficit” to a “language of difference.” This is done through subtle shifts in language, such as that in the current article. These linguistic choices serve to present ASDs or core attributes of ASD as a particular state of being that is not necessarily considered unwanted or in need of a cure. A discussion of preclinical detection can imply that preventative interventions must be used because autism itself is problemantic. However, in our discussion we advocate that early intervention can ultimately lead to promoting greater community involvement of autistic individuals rather than “normalizing” or eradicating autistic individuals. In both the clinical and research domains the goals and rationale of developing and implementing such technologies will need to be clearly and thoughtfully described.

While the 2014 US Patient Protection and Affordable Care Act (ACA) protects against discrimination of pre-existing conditions (29), it remains unclear how such a preclinical “diagnosis” or “assessment,” which is not a clinically confirmed diagnosis, would impact life- or long-term-care insurance policies. The US ACA states that all Marketplace insurance plans, must cover ASD screening, which usually consists of a behavioral checklist, for children at 18 and 24 months (30), yet it is unclear how the ACA would address the much more costly eye-tracking screening. In addition, only 41 states in the US have passed legislation requiring some level of insurance coverage for ASD services and therapies, but these policies rarely cover screenings (31). Many EU countries follow the recommendations the Royal College of Paediatrics and Child Health, the European Commission of Public Health, and the UK National Screening Committee (32, 33), which discourage community-wide screening (34). The World Health Organization’s resolution on ASD and recent meeting reports do not prioritize screening, focusing instead on increasing resources for autistic individuals and reducing stigma (21, 35). Even with national health care, most countries are not currently prepared to reimburse for infant or preclinical screens.

Stakeholders in the ASD community, including professionals, families, and diagnosed individuals, need to work with public policy makers, researchers, and clinicians to formulate strategies and regulations to determine when preclinical assessment should be performed and who is qualified to interpret and deliver such assessments (e.g., preclinical counselors, clinical psychologists, or primary care doctors). Because information about one’s brain health often feels especially identity forming (36), there must be safeguards for maintaining privacy of preclinical information. Without addressing these concerns, these tools, despite their enormous potential for providing opportunities for early intervention and substantial reduction of individual and societal healthcare costs, risk losing resources and public support to be fully developed and advanced.

## Author Contributions

Both JS and KR contributed to the research and writing of this article. JS focused on the areas concerning autism research and practice while KR focused on the neuroethical implications of the article. Both authors were equally involved in the development of this article.

## Conflict of Interest Statement

The authors declare that the research was conducted in the absence of any commercial or financial relationships that could be construed as a potential conflict of interest.
